# CD25 and TGF-β blockade based on predictive integrated immune ratio inhibits tumor growth in pancreatic cancer

**DOI:** 10.1186/s12967-018-1673-6

**Published:** 2018-10-25

**Authors:** Ning Pu, Guochao Zhao, Hanlin Yin, Jian-ang Li, Abulimiti Nuerxiati, Dansong Wang, Xuefeng Xu, Tiantao Kuang, Dayong Jin, Wenhui Lou, Wenchuan Wu

**Affiliations:** Department of General Surgery, Zhongshan Hospital, Fudan University, 180 Fenglin Road, Shanghai, 200032 People’s Republic of China

**Keywords:** Pancreatic ductal adenocarcinoma, Immunotherapy, Integrated immune ratio, Regulatory T cell, Program death receptor-1

## Abstract

**Background:**

The prognosis of pancreatic ductal adenocarcinoma (PDAC) remains poor due to the difficulty of disease diagnosis and therapy. Immunotherapy has had robust performance against several malignancies, including PDAC. In this study, we aim to analyze the expression of CD8 and FoxP3 on T lymphocytes and TGF-β expression in tumor tissues, and then analyze the possible clinical significance of these finding in order to find a novel effective immunotherapy target in PDAC using a murine model.

**Methods:**

A tissue microarray using patient PDAC samples was stained and analyzed for associations with clinicopathological characteristics. A preclinical murine model administrated with various immunotherapies were analyzed by growth inhibitor, flow cytometry, enzyme-linked immuno sorbent assay and immunohistochemistry.

**Results:**

The infiltrating FoxP3^+^ regulatory T cells (Tregs) in tumor tissues were associated with survival, while CD8^+^ tumor infiltrating lymphocytes (TILs) were not. Considering the drawbacks of these measure alone, the number of CD8^+^ and FoxP3^+^ T cells were combined to create a new estimated value—integrated immune ratio (IIR), which showed excellent validity in survival risk stratification. IIR was further verified as an independent prognostic factor according to multivariate analysis as well as TGF-β expression. Association between TGF-β expression and infiltrating Tregs was also verified. Then, in our preclinical murine model, CD25 and TGF-β combination blockade had a higher tumor growth inhibitor value. This combination therapy significantly depleted periphery and intra-tumor FoxP3^+^ Tregs while increasing intra-tumor CD8^+^ TILs levels compared to controls or anti-TGF-β monotherapy (*p *< 0.05). Anti-CD25 monotherapy alone also had the ability to deplete periphery and intra-tumor Tregs (*p *< 0.05). The excretion of intra-tumor IL-10, TGF-β was notably lower but higher IFN-γ excretion in this combination immunotherapy. Such combination immunotherapy was further confirmed to synergize with anti-PD-1 monotherapy to improve tumor growth inhibition and cure rates.

**Conclusions:**

The combination of CD25, TGF-β and PD-1 blockade plays a potentially effective role in inhibiting tumor formation and progression. Our results also provide a strong rational strategy for use of IIR in future immunotherapy clinical trials.

**Electronic supplementary material:**

The online version of this article (10.1186/s12967-018-1673-6) contains supplementary material, which is available to authorized users.

## Background

Pancreatic ductal adenocarcinoma (PDAC) is widely considered one of the most lethal digestive cancers and is the fourth common cause of cancer-related mortalities [1]. Surgical resection, neoadjuvant chemoradiotherapy, tumor vaccines or targeted therapy all show potential therapeutic efficacy for PDAC, but the 5-year overall survival remains dismally poor at about 8% [2, 3]. Nowadays, with the burgeoning trend of immunotherapy, many malignances are receiving good experimental and clinical efficacy with this novel therapy, including PDAC [4, 5].

PDAC has some unique characteristics including the existence of a dense stroma and tumor microenvironment full of immunosuppressive mediators. These unique characteristics made these tumors a dynamic entity both in terms of immune response and a solid barrier to drug penetration. Thus, the immune system has been thought to play an effective role in PDAC, which indicates that immunotherapy may be the most promising treatment for PDAC [6]. Furthermore, our previous studies have confirmed that there is a significant increase in infiltration of intra-tumor regulatory T cells (Tregs) in PDAC while a decrease of CD8^+^ tumor infiltrating lymphocytes (TILs) compared to surrounding tissue [7]. Tregs are generally considered to be one of the main obstacles to clinical efficacy of tumor immunotherapy, and have consistently confirmed their role in early establishment and progression of tumor [8]. Nowadays, many studies have attempted to target Tregs by modulating or depleting such cellular populations [9, 10]. The problem remains finding targets that are appropriate for Tregs interference and enhancement of CD8^+^ TILs, which is critical to the efficacy of immunotherapy.

CD25 is known as an interleukin-2 high-affinity receptor alpha chain (IL-2Ra), which is a surface marker of Tregs formerly used for identification and isolation before the discovery of transcription factor forkhead boxP3 (FoxP3) [11]. A large amount of pre-clinical studies in mice have confirmed that the anti-CD25 antibody clone PC-61 (rat IgG1, l) partially depletes Tregs in the peripheral lymphoid organs or blood, and inhibits tumor growth to improve survival in several cancers [8, 12]. Considering the intensive immunosuppression secondary to high-dose anti-CD25 antibodies, such monotherapies may not achieve the excellent effects [13]. Several previous clinical studies have demonstrated a variable impact on the vaccine-induced immunity and Tregs in combination with anti-CD25 treatment. However, no clear evidence exists for the effective decrease of Tregs in the tumor microenvironment from the limited indirect data assessing intra-tumor FoxP3 expression, and the anti-tumor efficacy appeared unsatisfactory in many previous studies with no apparent survival benefit [8].

The transforming growth factor beta (TGF-β) is known as a crucial regulatory signal for Tregs, by inducing the expression of FoxP3. In contrast, it is also one of the major functional cytokine excreted by Tregs and plays a vital role in cell differentiation and inflammation; functions which have been showed promote tumor progression [14–16]. In Smad4-inactivated PDAC, TGF-β signal pathway regulated a number of biological functions involving in extracellular matrix deposition, immunosuppression, etc. [17]. In addition, TGF-β was regarded as a key point in communication between pancreatic stellate cells and cancer cells, which contributed to the immunosuppressive microenvironment and tumor progression [18]. Enhanced anti-cancer activities by blocking TGF-β or its receptors had been verified in several pre-clinical cancer models. However, TGF-β blockade did not have promising results in these trials and further combination could be considered [19].

Program death receptor-1 (PD-1) is thought to be expressed on activated T cells [20, 21]. Receptor binding to its ligand (PD-L1) may lead to T cell exhaustion, or anergy, and impairs anti-tumor immune responses. PD-L1 is upregulated in many malignancies and associated with poor prognosis. Therefore, this study aimed to explore the presence of CD8^+^ and FoxP3^+^ infiltrating T cells and TGF-β expression in tumor tissues and analyze their clinical significance. Furthermore, the efficacy of enhancing tumor immune response and suppressing tumor growth with a combination of CD25 and TGF-β blockade was evaluated in a pre-clinical PADC murine model, as well as its synergistic effect with anti-PD-1 therapies.

## Materials and methods

### Patients and treatment

This study was approved by the Ethics and Research Committees of Zhongshan Hospital, Fudan University, China. The participant samples for tissue microarray (TMA) were selected from all patients registered at our hospital with pancreaticoduodenectomy (PD) or distal pancreatectomy (DP) between September 18, 2012, and May 4, 2016, with complete survival data. Formalin-fixed, paraffin-embedded tissue blocks from resected PDAC were made and 3.0 mm diameter tissue cylinders were punched from typical tissue areas. The histopathological type of the tissue was confirmed by experienced pathologists. The TMAs included detailed clinicopathological data, including patient age, sex, primary site, differentiation grade, T stage, lymph node metastasis, nervous invasion, vascular invasion, preoperative blood examinations and follow-up data (ended in December, 2016). In total, 90 patients were included in this study, 56 males and 34 females, with a median age of 64.5 years (ranging from 43 to 83 years). The median overall survival (OS) was 18 months (ranging of 1–42 months). More detailed patient information is listed in Table 1.

**Table 1:** Univariate and multivariate analysis of prognostic factors associated with overall survival

Characteristics	Patients	Overall survival			
		Univariate analysis *p* value	Multivariate analysis *p* value	Multivariate HR	95% confidence interval (CI)
Total	90				
Gender					
Male/female	56/34	0.782	NA		
Age (years)					
< 70/≥ 70	64/26	0.261	NA		
Primary site					
Head/body or tail	52/38	0.397	NA		
Differentiation					
I/II/III	1/28/61	0.303	NA		
T classification					
≤ 4 cm/> 4 cm	66/24	*0.039*	*0.047*	1.737	1.006–2.998
N classification					
N0/N1–2	53/37	*0.045*	*0.037*	1.741	1.035–2.927
Fibrinogen					
≤ 400/> 400 mg/dL	75/15	0.319	NA		
CA19-9					
< 37/≥ 37 U/L	22/68	0.207	NA		
CEA					
< 5/≥ 5 ng/mL	68/22	0.619	NA		
TBIL					
≤ 20.4/> 20.4 μmol/L	56/34	0.237	NA		
Albumin					
< 35/≥ 35 g/L	16/74	0.805	NA		
ALT					
≤ 35/> 35 U/L	50/40	0.441	NA		
AST					
≤ 40/> 40 U/L	54/36	0.672	NA		
GGT					
≤ 60/> 60 U/L	45/45	0.766	NA		
ALP					
≤ 125/> 125 U/L	54/36	0.588	NA		
LDH					
≤ 245/> 245 U/L	76/14	0.336	NA		
Glucose					
≤ 5.6/> 5.6 mmol/L	38/52	0.090	NA		
TGF-β					
Low/high	50/40	*< 0.001*	*0.001*	2.453	1.410–4.266
IIR					
Low/high	30/60	*0.006*	*0.032*	2.004	1.063–3.779

Italic values indicate significance of* p* value (*p*<0.05)

### Pancreatic cancer cells

Mice pancreatic duct adenocarcinoma cells (Panc02, a kind gift from Johns Hopkins Hospital, Baltimore, USA) were maintained at 37 °C and 5% CO_2_ in a medium containing RPMI-1640 medium (Gibco, Grand Island, New York, USA) supplemented with 10% fetal calf serum (Gibco), 100 U/mL penicillin and 100 U/mL streptomycin (Gibco).

### Animal experiments

Seventy-six female, 6–8-week-old C57BL/6 mice (Shanghai Slac Laboratory Animal Company, Shanghai, China) were maintained under specific pathogen free conditions. All animal experimental procedures were performed by following the China Animal Welfare Guidelines. The study protocol was approved by our Institutional Animal Care and Use Committee at Zhongshan Hospital, Fudan University, Shanghai, China.

Among the total group, forty mice were divided into four groups and were subcutaneously inoculated with 2 × 10^6^ Panc02 cells, each group receiving CD25 antibodies (Clone PC61) (Bio X Cell, West Lebanon, New Hampshire, USA) and TGF-beta antibodies (Clone 1D11) (Bio X Cell, West Lebanon, New Hampshire, USA) when tumor sizes were equal to 2 mm. Phosphate buffered solution (PBS), 50 μg CD25 antibodies, 100 μg TGF-beta antibodies and 50 μg CD25 antibodies combined with 100 μg TGF-beta antibodies in 200 μL PBS were injected intraperitoneally 3 times a week for 1 week into each subgroup, respectively. All injection doses were referred according to previous studies [8, 19, 22]. Mice in this experimental group were then sacrificed and the tumor and spleen tissue as well as blood were harvested for further experimentation.

The other mice (n = 36) were subcutaneously injected with 2 × 10^6^ Panc02 cells and were randomized into 7 groups receiving different treatments: (1) PBS control; (2) CD25 antibodies 50 μg; (3) TGF-beta antibodies 100 μg; (4) PD-1 antibodies 100 μg; (5) CD25 antibodies 50 μg and TGF-beta antibodies 100 μg; (6) CD25 antibodies 50 μg combined with TGF-beta antibodies 100 μg and PD-1 antibodies 100 μg. These treatments were administered respectively twice a week for 3 weeks beginning 3 days after inoculation (Figs. 4a and 5a). Tumor length and width were measured once weekly. Tumor volume was calculated with the formula: tumor volume = (length × width^2^)/2. The tumor growth inhibition (TGI) value (percent) was considered to be an indication of antitumor effectiveness. This value was calculated as TGI = (TVC − TVT)/TVC × 100%, where TVC is the mean tumor volume of the control group and TVT is the mean tumor volume of the treatment group, on a given experimentation day.

### Flow cytometry

Tumor tissues were digested with the Tumor Dissociation Kit (Miltenyi, Auburn, Washington, USA) according to the manufacturer’s instructions for a single-cell suspension. The collected spleen and blood were lysed with lysing buffer (BD Bioscience, Franklin Lakes, New Jersey, USA) and the cell pellets were stained with CD3-FITC (Biolegend, San Diego, California, USA), CD8a-PE-Cy7 (BD Bioscience), CD4-PerCP-Cy5.5 (BD Bioscience), FoxP3-Alex Fluorescence 647 (BD Bioscience) and PD-1-PE (BD Bioscience). The population of CD4^+^, CD8^+^ and PD-1^+^ T cells and CD4^+^FoxP3^+^ Tregs in CD3^+^ T lymphocytes were analyzed with FACS Aria II flow cytometer (BD Bioscience).

### Enzyme-linked immuno sorbent assay (ELISA)

A tumor tissue homogenate was prepared with RIPA containing 1% PMSF. The concentrations of TGF-β, IL-10 and IFN-γ in the tissue homogenate were determined by ELISA kit (R&D Systems, Tustin, California, USA), according to the manufacturer’s instructions.

### Immunohistochemistry (IHC)

Formalin-fixed, paraffin-embedded tumor specimens were histologically observed by HE staining. Representative thymus tissues were selected as positive controls and slides incubated without primary antibodies were used as negative controls. Rabbit anti-mouse FoxP3 (R&D Systems), CD8 (BD Bioscience) monoclonal antibodies, PD-1 (Cell Signaling Technology, Massachusetts, USA) and PD-L1 (Boster, Wuhan, China) were obtained and IHC of tumor tissues was performed as described previously [7].

### Tissue microarray and scoring

Surgical specimens were formalin-fixed, paraffin-embedded and histologically examined with HE staining. Representative areas of tissues were selected for analysis, precluding from necrotic and hemorrhagic tissue. Different tissue microarray blocks of 3 mm diameter were constructed and mounted on glass slides by sequencing. Rabbit anti-human FoxP3 (R&D Systems), mouse anti-human CD8 (Abcam, Cambridge, UK) and rabbit anti-human TGF-β (Abcam) monoclonal antibodies were used. IHC of serial tissue microarrays was performed as described previously.

The number of Foxp3^+^ Tregs and CD8^+^ TILs and the expression of TGF-β were calculated independently by two authors (P.N. and Z.GC.) who were blinded to the section treatment. The cell counts were executed using at least three independent 400× high-power fields (HPF). The influence of a small versus large number of intra-tumor Foxp3^+^ Tregs and CD8^+^ TILs was then evaluated. Patient micro-array data were classified into either high-count group or low-count group according to their cut-off values. The novel measure, integrated immune ratio (IIR), was formulated with the equation: IIR = (number of FoxP3^+^ Tregs)^2^/(number of CD8^+^ TILs). Immunoreactivity score of TGF-β in tumor tissues was derived by multiplying the intensity of IHC staining, the percentage of immunoreactive cells ranged from 0 to 12, and the average score on each slide (three images) was used to represent a particular sample. Samples with a final staining score of < 6 were classified to be low expression, while those with score of ≥ 6 were considered as high expression.

### Statistical analyses

The categoric values of experiments are presented as mean ± standard deviation $\left( {{\bar{\text{x}}} \pm {\text{s}}} \right).$ Statistical analyses were performed with SPSS 21.0 software (IBM, Almon, New York, USA) and the cut-off values for positive CD8^+^ and FoxP3^+^ cell numbers were determined through receiver operating characteristic curve (ROC) analysis. The relationships between clinicopathological variables and CD8, FoxP3 and TGF-β expression were analyzed by Pearson Chisquared test, Fisher’s exact test or Mann–Whitney U test, as appropriate. Kaplan–Meier analysis was used to create a survival curve in log-rank test. Cox proportional hazards regression model was used for univariate and multivariate analyses. Data with normal distribution were assessed with one-way ANOVA analysis and the LSD or Tamhane method were used for further analyses. Finally, tumor growth curves were depicted with GraphPad Prism 6.0 (GraphPad Software, San Diego, California, USA) and *p *< 0.05 was considered statistically significant.

## Results

### FoxP3^+^ and CD8^+^ Infiltrating T cells and TGF-β expression in PDAC TMA and staining features

**Fig. 1:**
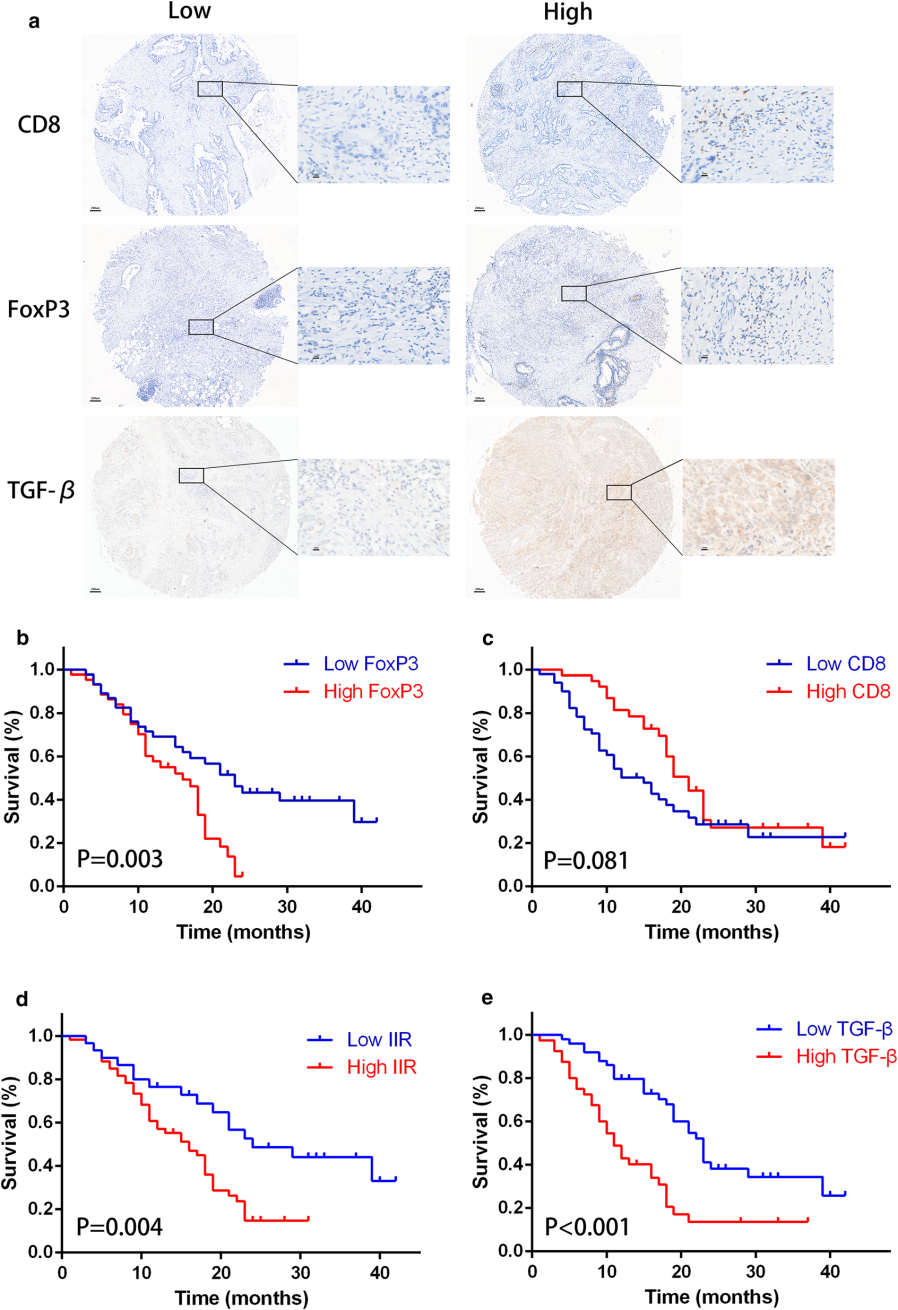
The immunohistochemistry of CD8 and FoxP3 in tissue microarray and its associated survival. **a** The representative low and high expression of CD8, FoxP3 and TGF-β (×40 low-power and ×400 high-power fields respectively). Kaplan–Meier survival curves of **b** FoxP3, **c** CD8, **d** IIR and **e** TGF-β was showed and analyzed using Log-rank test

The results of IHC stain are showed in Fig. 1a and summarized in Table 1. At low (40×) and high (400×) magnification, CD8 was located on T cell membranes, while Foxp3 was distributed in the T cells nucleus. TGF-β was mainly expressed in the cytoplasm. Through analysis, CD8 was highly expressed in 43.3% (39/90) of PDAC samples, FoxP3 was highly expressed in 48.9% (44/90), while TGF-β in 44.4% (40/90).

### Relationship between FoxP3^+^ Tregs or CD8^+^ TILs and TGF-β expression

The mean number of positive CD8^+^ TILs and FoxP3^+^ Tregs was 39.2 ± 36.9 and 8.7 ± 10.7 respectively. The most suitable cut-off value was 32.5 for CD8^+^ TILs and 5.5 for FoxP3^+^ Tregs. The relationships between FoxP3^+^ Tregs or CD8^+^ TILs in intra-tumor tissues and TGF-β staining or other clinicopathological characteristics were shown in Additional file 1: Table S1. Infiltrating FoxP3^+^ Tregs were significantly associated with the albumin (*p *= 0.004), glucose levels (*p *= 0.017) and TGF-β expression (*p *= 0.002), but there was no significant correlation with other factors. Additionally, no positive results were found for CD8^+^ TILs (all *p* > 0.05).

### Prognostic impact of TGF-β expression, FoxP3^+^ Tregs, CD8^+^ TILs and novel evaluation index—IIR

The 1- and 3-year OS rates of all 90 patients who underwent pancreatectomy for PDAC were 63.7% and 21.9% respectively. The prognostic impact of intra-tumor FoxP3^+^ Tregs and CD8^+^ TILs on OS was analyzed by univariate and multivariate analyses. In the Kaplan–Meier curve analysis, FoxP3 expression Tregs were significantly considered to be a prognostic factor correlated with OS (Fig. 1b, *p *= 0.003), while presence of intra-tumor CD8^+^ TILs showed no prognostic significance for OS (Fig. 1c, *p *= 0.081).

According to the survival curves of FoxP3^+^ Tregs and CD8^+^ TILs, the difference between high and low FoxP3 expression groups could not be discerned at the beginning of the curves. In addition, there was no significant difference in high and low CD8 expression groups and overlaps were found in the latter part of the survival curves. Therefore, we integrated the number of CD8^+^ TILs and FoxP3^+^ Tregs into one novel formulation, called IIR. The Kaplan–Meier analysis of IIR had statistical significance and superior separated risk stratification (*p *= 0.004) (Fig. 1d). In addition, TGF-β was significantly found to be a prognostic factor (*p *< 0.001) and patients with higher TGF-β expression were trend to sustain a shorter survival (11.0 vs. 23.0 months) (Fig. 1e). Furthermore, all significant risk factors were used into multivariate analysis with a Cox proportional hazards model (Table 1). Results of these analyses showed that T classification (*p *= 0.047), N classification (*p *= 0.037), TGF-β expression (*p *= 0.001) and IIR (*p *= 0.032) all remained independently and negatively associated with OS. The median OS of patients with higher IIR was significantly lower than that of patients with lower IIR (16.0 vs. 23.0 months, respectively). The 1-year OS rate of patients with higher IIR was 50.3%, while those with lower IIR was 80.0%.

### CD25 and TGF-β blockade reduces both intra-tumor and peripheral FoxP3^+^ Tregs

**Fig. 2:**
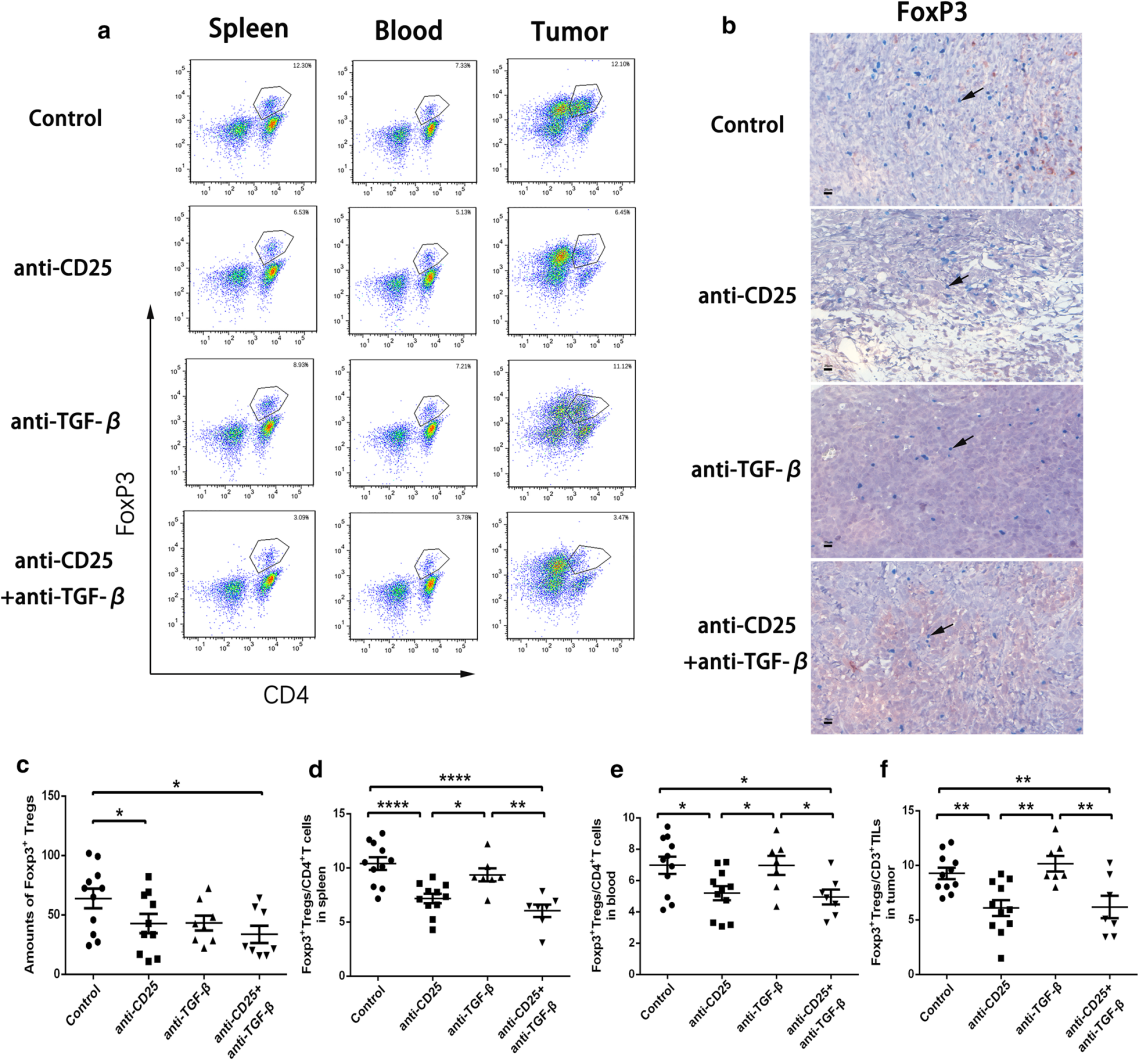
Combination therapy of anti-CD25 and anti-TGF-β depletes Tregs in PDAC murine models. **a** Flow cytometry gating schema and density plots for CD3^+^CD4^+^FoxP3^+^ Tregs. **b** FoxP3 expression in tumor tissues under different therapies. IHC data showing **c** the amounts of FoxP3^+^ Tregs. Flow cytometry data showing the percentage of Tregs among CD4^+^ T cells in **d** spleen and **e** blood, and **f** the percentage of Tregs among CD3^+^ T cells in tumor. *< 0.05, **< 0.001, ****< 0.0001

Owning to the relationships and functions of CD8^+^ TILs, FoxP3^+^ Tregs and TGF-β in tumor tissues, we attempted to block both CD25 and TGF-β in mice models. To investigate the changes of lymphocyte cohorts after therapy, spleen and tumor tissue as well as blood were harvested for flow cytometry analysis. Given the immune suppression of Tregs in pancreatic cancer, we examined whether the immunotherapies affected intra-tumor or peripheral Tregs. On account of the blockade of CD25 receptor, we identified CD3^+^CD4^+^FoxP3^+^ as Tregs (Fig. 2a). In the analysis of spleen single-cell suspension or blood, either CD25 blockade alone or combination therapies saw a lower percentage of Tregs among CD4^+^ T cells than TGF-β neutralization alone or control groups (Fig. 2d, e). However, TGF-β neutralization alone did not make any significant changes to Tregs population in the control group. In addition, there was still lack of statistical difference between combination therapies and CD25 blockade alone. In tumor tissues, the percentage of Tregs among all CD3^+^ T cells showed the same downwards trend as the periphery (Fig. 2f). We further examined the FoxP3^+^ tumor infiltrating Tregs with IHC technology. Notably, combination anti-CD25 and anti-TGF-β therapy resulted in a significantly lower number of total tumor infiltrating Tregs compared to control group, which showed consistency to flow cytometry results (Fig. 2b, c). All these data revealed that the combination therapy reduced both intra-tumor and peripheral Tregs, which may improve physical immunity and local anti-tumor immune response.

### CD25 and TGF-β blockade enhances antitumor immune responses

**Fig. 3:**
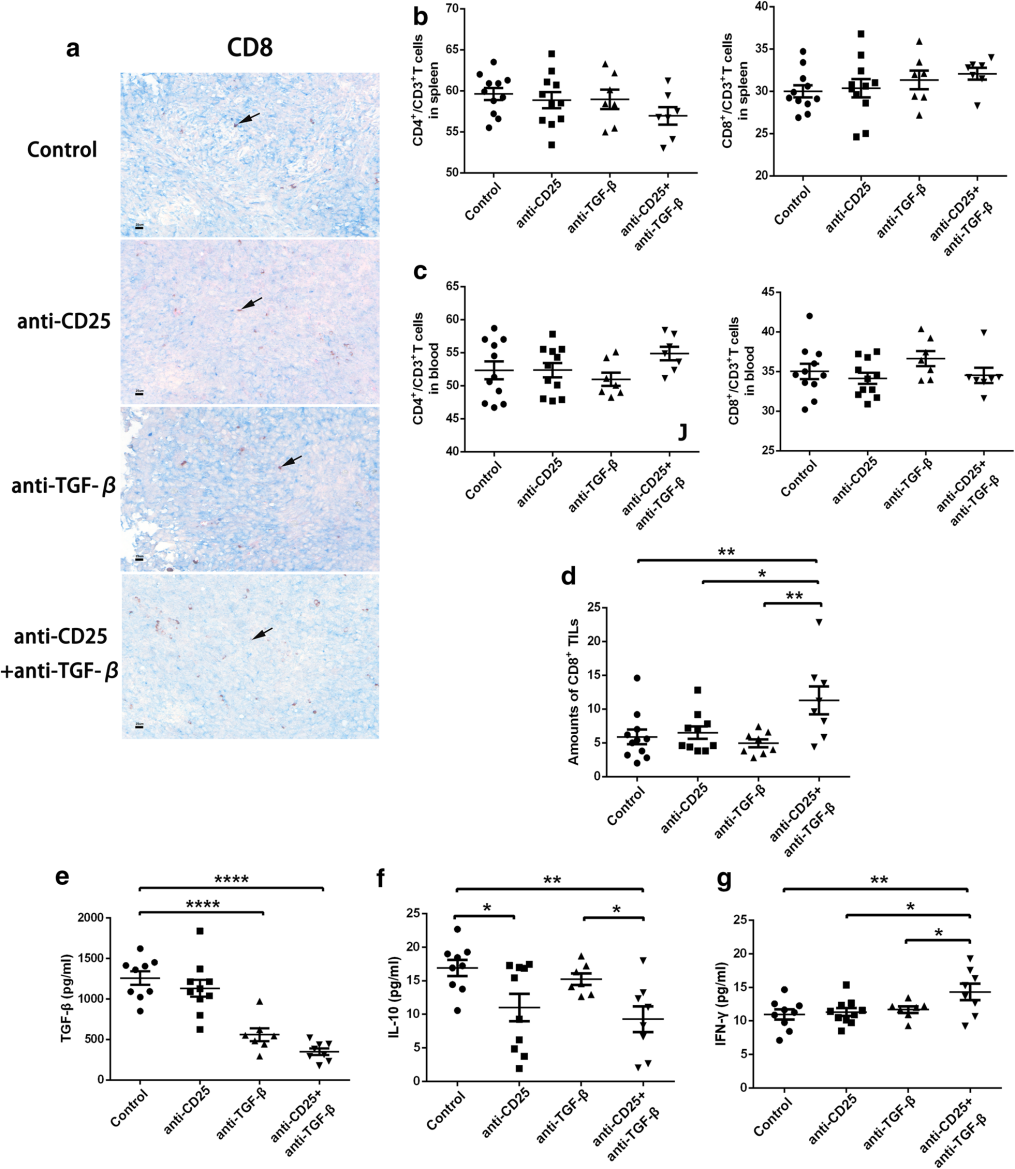
Combination therapy of anti-CD25 and anti-TGF-β enhances intra-tumor immune response. **a** CD8 expression in tumor tissues under different therapies. Flow cytometry data showing the percentage of CD4^+^ or CD8^+^ T cells among all CD3^+^ T cells in **b** spleen and **c** blood. **d** IHC data showing the amounts of CD8^+^ T cells in immunohistochemistry staining. The Elisa data showing the concentration of **e** TGF-β, **f** IL-10, and **g** IIFN-γ within tumor tissues. *< 0.05, **< 0.001, ****< 0.0001

Next, we examined the immune response affected by immunotherapies. We found that there was no significant difference in percentage of CD8^+^ and CD4^+^ T cells among all CD3^+^ T cell from the spleen or blood (Fig. 3b, c). As IHC analysis showed, anti-CD25 monotherapy or anti-TGF-β monotherapy had no significant effect on the absolute amount of CD8^+^ TILs. However, the combinational therapy significantly enhanced the absolute amount of CD8^+^ TILs in the tumor microenvironment compared to anti-CD25 monotherapy (11.3 vs. 6.5, *p *= 0.011), anti-TGF-β monotherapy (11.3 vs. 5.0, *p *= 0.002) and control group (11.3 vs. 5.9, *p *= 0.004). These results reflected the robust effect of combination therapy on mediating the immune response (Fig. 3a, d).

To further analyze immune function in the local tumor tissues, IFN-γ, TGF-β and IL-10 levels were analyzed in tissue homogenates. TGF-β and IL-10 are the main cytokines of Tregs that play a role in the immune suppression. Our previous results had showed the depletion of Treg numbers, so here we examined the depletion of Treg function. Through the analysis, we found TGF-β levels were significantly decreased with application of anti-TGF-β antibodies (Fig. 3e), while IL-10 levels were also declined significantly attenuated with the administration of anti-CD25 antibodies (Fig. 3f). Taken together, these results suggested that the combination of CD25 and TGF-β blockade may extremely inhibit the suppressive effect in the tumor microenvironment.

IFN-γ level represented the intensity of local anti-tumor immune response. Intriguingly, IFN-γ level in combination therapy was significantly higher than all other monotherapies or control group, which further demonstrated the superiority of the combination therapy (Fig. 3g). All of these results suggested CD25 allied with TGF-β blockade may deplete the tumor-associated suppressive cytokines and enhance the tumor-specific IFN-γ production to restrain tumor growth and progression.

### CD25 and TGF-β blockade delays tumor growth in murine tumor models

**Fig. 4:**
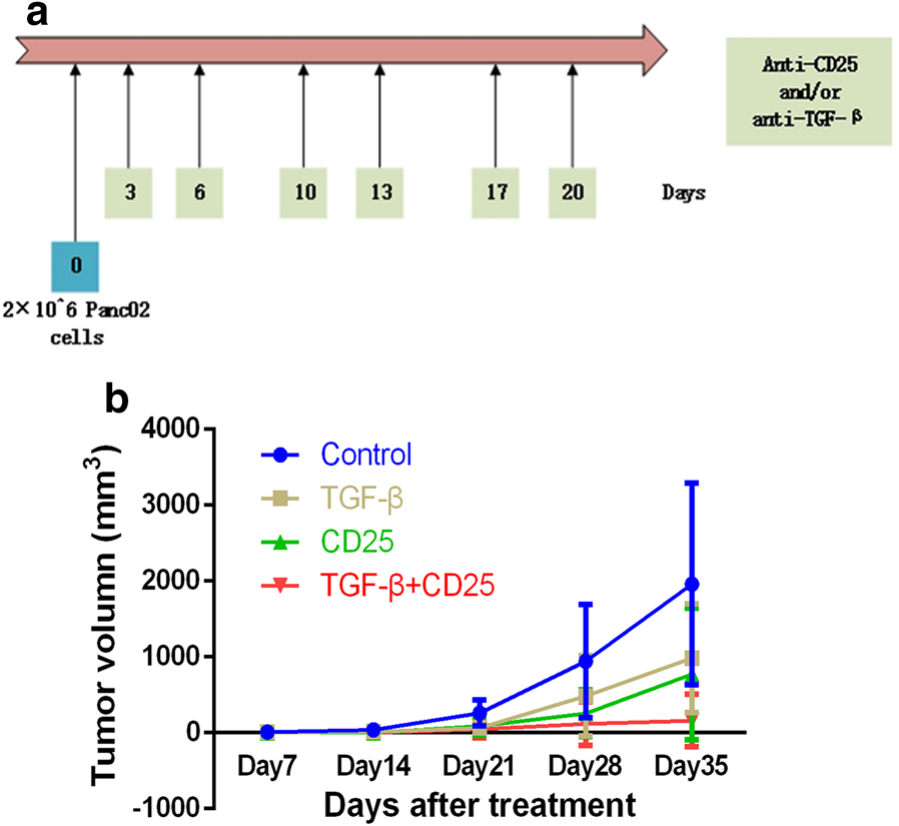
Combination therapy of anti-CD25 and anti-TGF-β improves clinical outcomes in PDAC murine models. **a** Schema of tumor implantation and treatment with anti-CD25 and anti-TGF-β. **b** Tumor growth curves of mice implanted with Panc02 tumor cells and administrated with anti-CD25 and/or anti-TGF-β

As shown in Table 2, tumor size on day 7, 14, 21, 28 and 35 with different immunotherapies was recorded. We found that in comparison to the PBS control group, changes of tumor volume on day 7 (*p *= 0.001), day 14 (*p *= 0.002), day 21 (*p *= 0.028), day 28 (*p *= 0.035) and day 35 (*p *= 0.008) with the combination therapy of anti-CD25 antibodies and anti-TGF-β antibodies were all significantly different (Additional file 2: Figure S1A). In addition, the group with CD25 blockade was also statistically different size tumors compared to the control group on day 7 (*p *= 0.012) and day 14 (*p *= 0.005). However, in comparison between other groups, there was a lack of significant difference. On endpoint observation, the TGI of anti-CD25 monotherapy, anti-TGF-β monotherapy and combination therapy group was 60.5, 49.8 and 91.8%, respectively (Fig. 4b). Our results revealed that the blockade of CD25 and TGF-β showed a superior effect on inhibition of tumor growth.

**Table 2:** The tumor volume with anti-CD25 and/or anti-TGF-β treatment in different periods

Cohorts	Number	Day 7 (*p* value)	Day 14 (*p* value)	Day 21 (*p* value)	Day 28 (*p* value)	Day 35 (*p* value)
Control	5	9.92 ± 5.82 (1)	38.30 ± 29.00 (1)	259.43 ± 171.47 (1)	942.92 ± 745.58 (1)	1962.19 ± 1326.31 (1)
Anti-TGF-β	5	5.19 ± 4.00 (0.142)	14.60 ± 9.08 (0.056)	63.57 ± 40.38 (0.053)	487.30 ± 530.90 (0.353)	984.64 ± 719.09 (0.211)
Anti-CD25	5	2.22 ± 2.90 (*0.012*)	3.97 ± 4.28 (*0.005*)	90.74 ± 123.26 (0.104)	257.59 ± 310.82 (0.104)	774.40 ± 867.07 (0.108)
Anti-TGF-β^+^ anti-CD25	6	0.01 ± 0.004 (*0.001*)	0.46 ± 0.85 (*0.002*)	48.31 ± 115.88 (*0.028*)	120.39 ± 288.12 (*0.035*)	160.81 ± 347.21 (*0.008*)

Italic values indicate significance of* p* value (*p*<0.05)

### PD-1 and PD-L1 expression in mice tumor specimens with CD25 and TGF-β blockade

The IHC staining results of mice treated tumor specimens showed that PD-1 expression among T lymphocytes in anti-CD25, anti-TGF-β or combination treatment was higher than the control cohort, while these three different treatment showed no significant difference in PD-1 expression. However, PD-L1 expression had no significant difference in separate four groups, and each group showed relative high expression (Additional file 3: Figure S2).

### Combination immunotherapy acts synergistically with anti-PD-1 therapy to eradicate pancreatic cancer

**Fig. 5:**
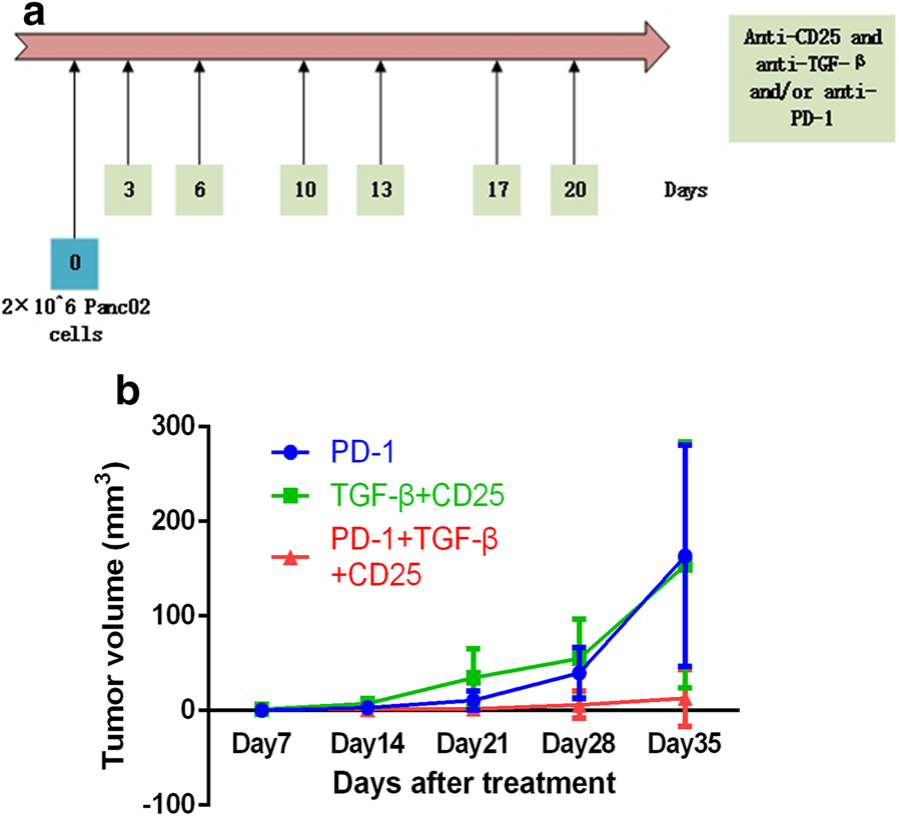
Combination therapy of anti-CD25 and anti-TGF-β was synergized with anti-PD-1 monotherapy. **a** Schema of tumor implantation and treatment with anti-PD-1 and anti-CD25 associated with anti-TGF-β. **b** Tumor growth curves of mice implanted with Panc02 tumor cells and administrated with anti-PD-1 and/or anti-CD25 associated with anti-TGF-β

Based on the above findings and the observed role of the PD-1-PD-L1 axis, we hypothesized that depletion of Treg numbers and PD-1 blockade-associated function may be significantly synergistic changes. As shown in Table 3, the anti-PD-1 monotherapy showed no advantage compared to combination therapy of CD25 and TGF-β blockade. Although, there was no significant difference between all groups earlier, mice receiving triple combination therapy displayed smaller tumor volume on day 35 (*p *= 0.043) compared to anti-PD-1 monotherapy. Additionally, on day 21 (*p *= 0.044), day 28 (*p *= 0.037), day 35 (*p *= 0.046) compared to combination therapy of CD25 and TGF-β blockade, triple combination therapy was more effective. To our surprise, the cure rate of the murine tumor models with triple combination therapy was up to 80% (4 out of 5), while only 20% (1 out of 5) cure rate with the anti-PD-1 monotherapy (Additional file 2: Figure S1B). Anti-PD-1 therapy in combination with CD25 and TGF-β blockade therefore appeared highly effective at limiting tumor proliferation and progression (Fig. 5b).

**Table 3:** The tumor volume with anti-PD-1 and/or anti-CD25 associated with anti-TGF-β treatment in different periods

Cohorts	Number	Day 7	Day 14	Day 21	Day 28	Day 35
Anti-PD-1	5	0.49 ± 0.45	3.02 ± 3.38	10.81 ± 9.82	39.71 ± 27.06	163.50 ± 117.02
Anti-TGF-β^+^ anti-CD25	5	1.56 ± 1.68	7.61 ± 7.08	34.94 ± 30.67	55.28 ± 41.42	129.87 ± 29.75
Anti-PD-1^+^ anti-TGF-β^+^ anti-CD25	5	1.93 ± 1.74	1.46 ± 2.09	1.84 ± 4.04	6.32 ± 14.13	13.31 ± 29.76
Multiple comparision *p* value	1 vs. 2	0.205	0.227	0.132	0.502	0.906
	1 vs. 3	0.139	0.411	0.114	0.050	*0.043*
	2 vs. 3	0.742	0.099	*0.044*	*0.037*	*0.046*

Italic values indicate significance of* p* value (*p*<0.05)

Then, T cell frequency, PD-1^+^ T cells and Treg frequency were analyzed by flow cytometry. The results showed that the PD-1^+^ T cell frequency under anti-PD-1 monotherapy or the combination therapy of CD25, TGF-β and PD-1 blockade was much lower than the combination therapy of CD25 and TGF-β blockade in the periphery (Additional file 4: Figure S3A), while total T cell frequency had no significant changes between these three groups (Additional file 4: Figure S3B and C). The peripheral Treg frequency under three combination therapy was significantly lower than that under anti-PD-1 monotherapy, but lack of statistical significance than that under two combination therapy. However, there was a trend in decrease of Treg counts among the three and two combination therapy (Additional file 4: Figure S3D).

## Discussion

In this study, infiltrating FoxP3^+^ Tregs and TGF-β expression in tumor tissues were found to be associated with survival. However, considering the considerable drawbacks of the two immune indicators alone, we merged the number of CD8^+^ TILs and FoxP3^+^ Tregs together to create a new estimated value—IIR, which showed excellent distinction in survival risk stratification. IIR was verified as an independent prognostic factor according to multivariate analysis, as well as TGF-β expression, T and N classification were.

CD8 to considered as glycoprotein heterodimer made up of alpha and beta chains covalently linked by a disulfide bond. The function of CD8 is to bind to a major histocompatibility complex class I molecule associated with the T cell receptor and stimulate its cytotoxic effect on cancer cells and induce a vital role in cell-mediated immunity [23]. Although many studies have shown patients with higher intra-tumor CD8^+^ TILs have superior survival in many malignancies [24–26], there was no significant difference in prognosis observed within our study probably due to the limited sample size used. In terms of the prognostic impact of FoxP3^+^ Tregs, observations in our study were in line with the previous studies that indicated a negative prognostic impact both in patients with PDAC and other malignant cancers [7, 27–29]. In addition, tumor immune response was considered to be a pivotal role in its local position, so a sole indicator could not completely indicate the real status of the immune response. IIR incorporated the positive indicator—CD8^+^ TILs with inhibitory FoxP3^+^ Tregs, which revealed better clinical benefit in predicting OS.

This report is the first examination the effect of combining CD25 and TGF-β blockade in pancreatic cancer treatment. Our results revealed that there were advantages to this treatment such as, partially depleting the number and function of FoxP3^+^ Tregs as well as enhancing the tumor-specific immune response, which could also synergize with anti-PD-1 therapy. Several previous studies have confirmed the function of CD25 blockade in depleting FoxP3^+^ Tregs in the tumor microenvironment, which activated FcγRs capable of inducing antibody dependent cellular cytotoxicity [8, 30, 31]. Considering that the depletion of tumor infiltrating FoxP3^+^ Tregs by anti-CD25 monotherapy resulted in only partial tumor control and large-dose possible complications [11], it was necessary to identify further interventions to increase clinical efficacy and tumor specific immune response.

Our data demonstrated that CD25 blockade was efficient in depleting intra-tumor or peripheral FoxP3^+^ Tregs, but not enhancing CD8^+^ TIL number and function. By contrast, the combination of CD25 and TGF-β blockade had superior effects on tumor control, CD8^+^ T cell infiltration and tumor immune intensity. Importantly, the combination therapy not only decreased the number of Tregs, but also decreased the functional expression of immune suppressive Tregs in the tumor microenvironment.

Previous studies have shown that expression of SMAD4 SNP or TGF-βR2/SMAD4 tumor protein in relation to the TGF-β pathway in pancreatic cancer can be utilized in clinical trials of targeted therapies [17]. The TGF-β signaling pathway promoted physiological processes such as cell growth, proliferation, differentiation, fibrosis, and scar formation [22]. It played a vital role in tumor progression through angiogenesis induction and immune suppression by inhibiting Tregs apoptosis. This pathway also showed particularly potential as a therapeutic target and a prognostic factor being part of TGF-β/Smad4 signaling in the pathogenesis of PDAC [15, 32]. In addition, the correlation between TGF-β and Tregs was also confirmed in hepatic carcinoma patients and the expression of both TGF-β and IL-10 was shown to be associated with hepatic carcinoma progression [16], as well as PDAC [33]. Anti-TGF-β monotherapy had an effect on increasing CD8^+^ TILs and their excretion of IFN-γ, however, there was no significant variation in Tregs. Finally, researchers managed to combine the TGF-β blockade with a GM-CSF secreting allogeneic pancreas tumor vaccine (GVAX) to effectively decrease levels of tumor infiltrating Tregs [19]. Our study also found that higher TGF-β expression was highly associated with the number of infiltrating FoxP3^+^ Tregs and longtime survival, which led our group to conceive the combination immunotherapy examined in this study.

Although anti-CD25 monotherapy demonstrated little effect on tumors at the beginning of treatment, the tumor volume growth accelerated late in the treatment course, therefore demonstrating that targeting CD25 was not sufficient to control or cure the tumor. However, the combination of CD25 and TGF-β blockade treatment led to improve tumor control and microenvironment regulation compared to anti-CD25 monotherapy or anti-TGF-β monotherapy. Only approximately 33.3% of mice were cured with the combination of CD25 and TGF-β blockade, which was still an unsatisfactory therapeutic result. There was lack of obvious advantages compared to previous reports of the cure rate for combination GVAX and anti-TGF-β monotherapy was about 30% and further association with anti-PD-1 monotherapy raised the rate to 50% [19]. Anti-PD-1 monotherapy has become a dominant treatment paradigm for multiple solid malignant cancers with response rates ranging from 20 to 30% [34]. Through our observation from the PD-1/PD-L1 staining of treated mice specimens, we believe, both Tregs and PD-1 pathways should be targeted to maximize the effect of such treatments. Importantly our data showed this triple combination treatment significantly contributed to tumor growth suppression with up to 80% cure rate. Nevertheless, other potential immunosuppressive pathways remain to be explored and identified to further improve the clinical efficacy and outcome in patients with PDAC.

Our preclinical murine PDAC model concentrated on tumor growth after inoculation, which was similar to the situation of PADC patients after radical resection. Our results suggest the combination of CD25, TGF-β and PD-1 effectively inhibited tumor formation and progression via depleting tumor infiltrating FoxP3^+^ Tregs and enhancing anti-tumor immune response on the basis of IIR. Our clinicopathological analysis and preclinical studies indicate immunotherapy may be beneficial in the treatment of PDAC. Further evaluation in clinical trials may also be applicable in order to confirm its clinical value.

However, there were still some limitations in our study. Firstly, limited tumor tissues from patients were collected from a single center in China and the cut-off value of IIR may not be appropriate to other studies, so a multicenter and large-scale research should be preferred to verify our results. Secondly, this was just a preclinical research and lack of further molecular mechanism, so further clinical trials and mechanism research were needed to confirm our findings.

## Conclusions

The number of CD8^+^, FoxP3^+^ T cells and TGF-β expression in PDAC were potentially associated with patients’ OS. IIR may be showed as an excellent index for validity in survival risk stratification. Thus, in terms of its specific microenvironment, the combination of CD25, TGF-β and PD-1 blockade may play a potentially effective role in inhibiting tumor formation and progression. The results also provide a strong rational strategy in future immunotherapy clinical trials.

## Additional files

**Additional file 1: Table S1.** Relationships between FoxP3^+^ and CD8^+^ infiltrating T cells and clinicopathological characteristics.

**Additional file 2: Figure S1.** The tumor volume after treatments. (A) Comparing the treatment effects with anti-CD25 and anti-TGF-β. (B) Comparing the treatment effects with anti-CD25, anti-TGF-β and anti-PD-1.

**Additional file 3: Figure S2.** The PD-1/PD-L1 expression in tumor tissue after treatments with anti-CD25 and anti-TGF-β.

**Additional file 4: Figure S3.** The changes of peripheral T cell cohorts and PD-1 expression after treatments with anti-CD25, anti-TGF-β and anti-PD-1. Flow cytometry gating schema and density plots for (A) CD3^+^CD45^+^PD-1^+^ T cells, (B) CD3^+^CD45^+^ T cells, (C) CD3^+^CD4^+^ or CD3^+^CD8^+^ T cells and (D) CD3^+^CD4^+^FoxP3^+^ Tregs.

## Abbreviations

PDAC: pancreatic ductal adenocarcinoma
TGF-β: transfer growth factor beta
PBS: phosphate buffered solution
IL: interleukin
IFN-γ: interferon-γ
TILs: tumor-infiltrating lymphocytes
Treg: regulatory T cell
FoxP3: transcription factor forkhead boxP3
OS: overall survival
TMA: tissue microarray
ELISA: enzyme-linked immuno sorbent assay
IHC: immunohistochemistry
HPF: high-power field
