# Supplementary material for: CD25 and TGF-β blockade based on predictive integrated immune ratio inhibits tumor growth in pancreatic cancer
Source: J Transl Med. 2018 Oct 25;16:294. doi: 10.1186/s12967-018-1673-6 (PMC6203282; doi:10.1186/s12967-018-1673-6)
Supplement: Supplementary file 1 — Additional file 1: Table S1. Relationships between FoxP3+ and CD8+ infiltrating T cells and clinicopathological characteristics. [file 12967_2018_1673_MOESM1_ESM.docx]

**Table S1. Relationships between FoxP3^+^ and CD8^+^ infiltrating T cells and clinicopathological characteristics.**

| **Characteristics** | **Low FoxP3** | **High FoxP3** | ***P* value** | **Low CD8** | **High CD8** | ***P* value** |
| --- | --- | --- | --- | --- | --- | --- |
|  | **(n=46)** | **(n=44)** |  | **(n=51)** | **(n=39)** |  |
| **Gender** |  |  | 0.787 |  |  | 0.578 |
| Male/ Female | 28/18 | 28/16 |  | 33/18 | 23/16 |  |
| **Age (years)** |  |  | 0.893 |  |  | 0.080 |
| <70/ ≥70 | 33/13 | 31/13 |  | 40/11 | 24/15 |  |
| **Primary site** |  |  | 0.501 |  |  | 0.135 |
| Head/ Body or tail | 25/21 | 27/17 |  | 26/26 | 26/13 |  |
| **Differentiation** |  |  | 0.132 |  |  | 0.494 |
| I/ II/ III | 1/18/27 | 0/10/34 |  | 1/14/36 | 0/14/25 |  |
| **T classification** |  |  | 0.727 |  |  | 0.773 |
| ≤4cm/ > 4cm | 33/13 | 33/11 |  | 38/13 | 28/11 |  |
| **N classification** |  |  | 0.696 |  |  | 0.379 |
| N0/N1-2 | 28/18 | 25/19 |  | 28/23 | 25/14 |  |
| **Fibrinogen** |  |  | 0.346 |  |  | 0.775 |
| ≤400/ > 400 mg/dl | 40/6 | 35/9 |  | 42/9 | 33/6 |  |
| **CA19-9** |  |  | 0.065 |  |  | 0.817 |
| < 37/≥37 U/L | 15/31 | 7/37 |  | 12/39 | 10/29 |  |
| **CEA** |  |  | 0.389 |  |  | 0.817 |
| < 5/ ≥ 5 ng/mL | 33/13 | 35/9 |  | 39/12 | 29/10 |  |
| **TBIL** |  |  | 0.142 |  |  | 0.320 |
| ≤ 20.4/>20.4 μmol/L | 32/14 | 24/20 |  | 34/17 | 22/17 |  |
| **Albumin** |  |  | **0.004** |  |  | 0.250 |
| < 35/ ≥ 35 g/L | 3/43 | 13/31 |  | 7/44 | 9/30 |  |
| **ALT** |  |  | 0.300 |  |  | 0.116 |
| ≤ 35/ > 35 U/L | 28/18 | 22/22 |  | 32/19 | 19/21 |  |
| **AST** |  |  | 0.302 |  |  | 0.297 |
| ≤ 40/ > 40 U/L | 30/16 | 24/20 |  | 33/18 | 21/18 |  |
| **GGT** |  |  | 1.000 |  |  | 0.832 |
| ≤ 60/ > 60 U/L | 23/23 | 22/22 |  | 26/25 | 19/20 |  |
| **ALP** |  |  | 0.547 |  |  | 0.543 |
| ≤ 125/ > 125 U/L | 29/17 | 25/19 |  | 32/19 | 22/17 |  |
| **LDH** |  |  | 0.210 |  |  | 0.584 |
| ≤245/ >245 U/L | 41/5 | 35/9 |  | 44/7 | 32/7 |  |
| **Glucose** |  |  | **0.017** |  |  | 0.818 |
| ≤ 5.6/ >5.6 mmol/L | 25/21 | 13/31 |  | 21/30 | 17/22 |  |
| **TGF-β**  Low/High | 33/13 | 17/27 | **0.002** | 27/24 | 23/16 | 0.568 |
