# Supplementary figures and images for: CD25 and TGF-β blockade based on predictive integrated immune ratio inhibits tumor growth in pancreatic cancer
Source: J Transl Med. 2018 Oct 25;16:294. doi: 10.1186/s12967-018-1673-6 (PMC6203282; doi:10.1186/s12967-018-1673-6)

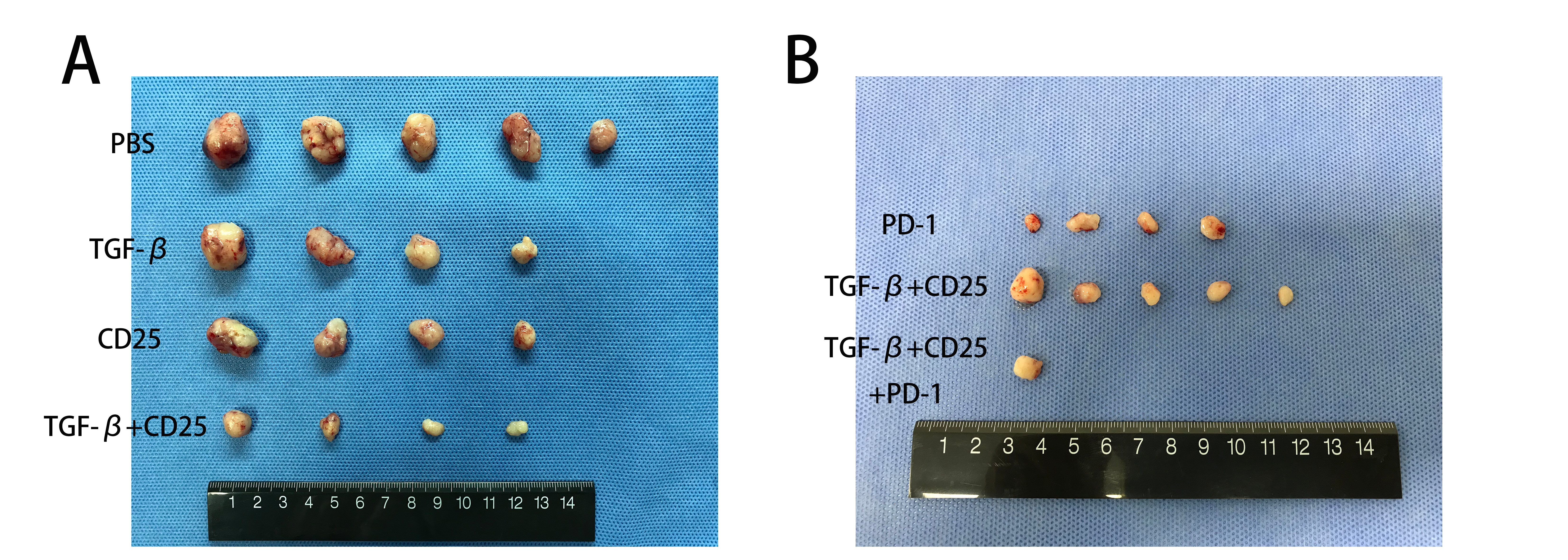

Supplement: Supplementary file 2 — Additional file 2: Figure S1. The tumor volume after treatments. (A) Comparing the treatment effects with anti-CD25 and anti-TGF-β. (B) Comparing the treatment effects with anti-CD25, anti-TGF-β and anti-PD-1. [file 12967_2018_1673_MOESM2_ESM.tif]

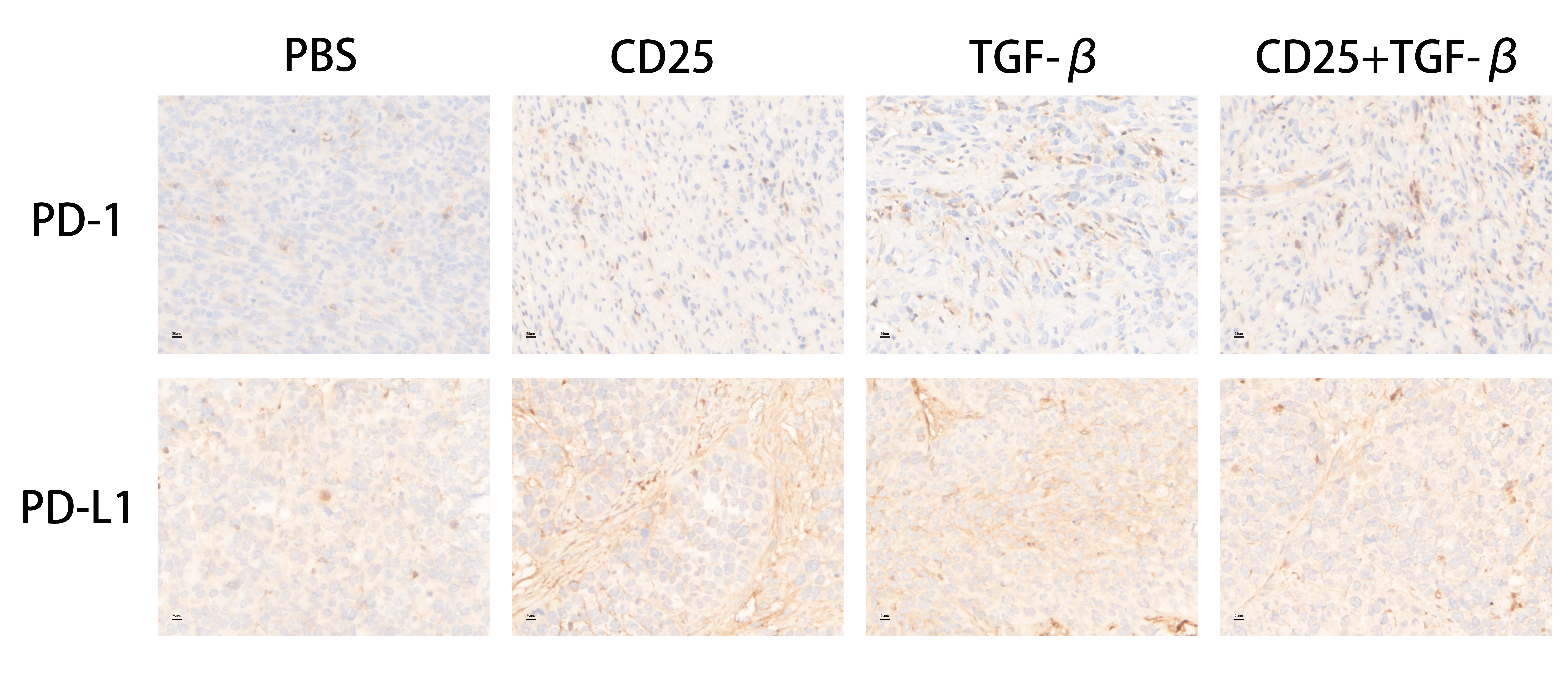

Supplement: Supplementary file 3 — Additional file 3: Figure S2. The PD-1/PD-L1 expression in tumor tissue after treatments with anti-CD25 and anti-TGF-β. [file 12967_2018_1673_MOESM3_ESM.tif]

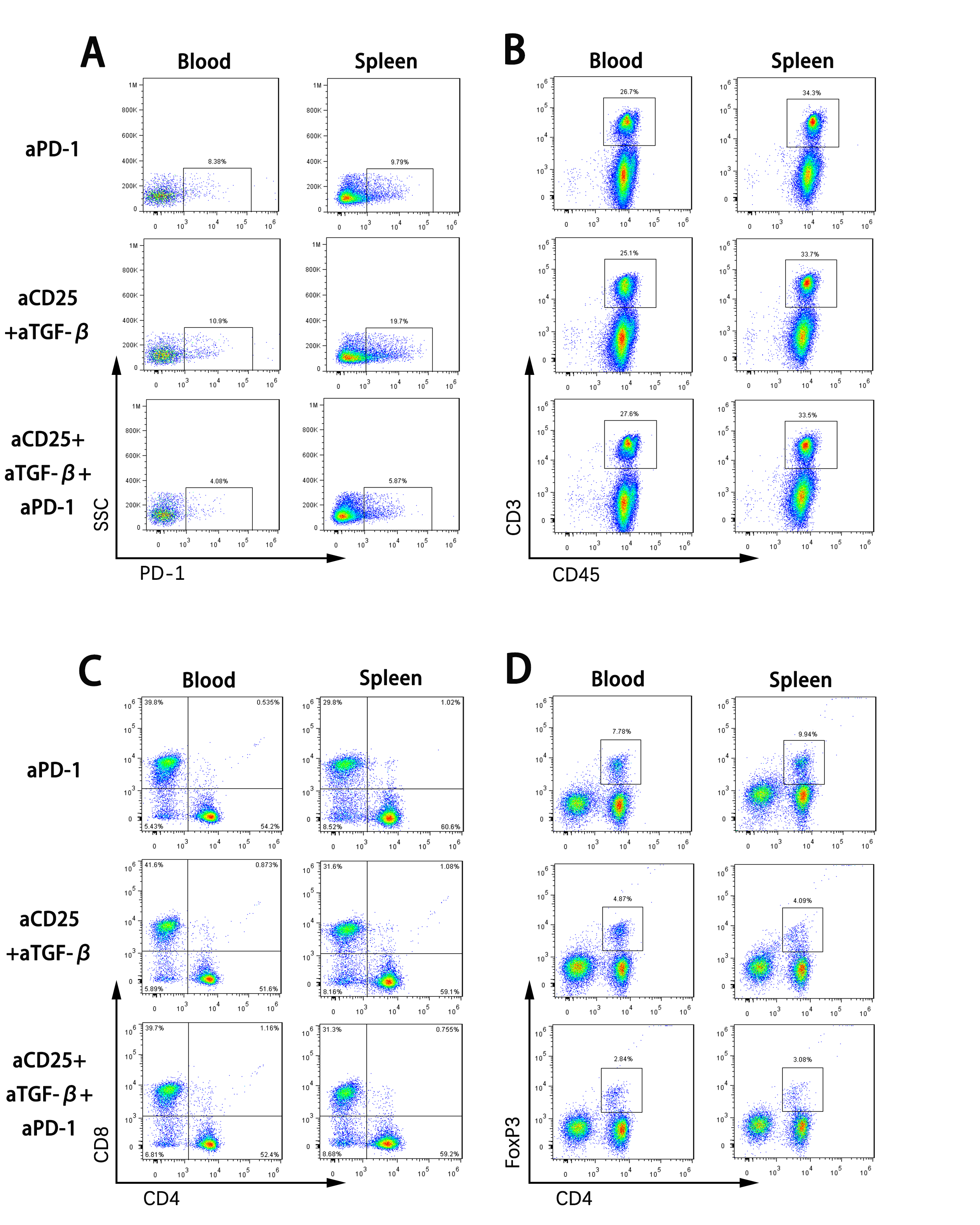

Supplement: Supplementary file 4 — Additional file 4: Figure S3. The changes of peripheral T cell cohorts and PD-1 expression after treatments with anti-CD25, anti-TGF-β and anti-PD-1. Flow cytometry gating schema and density plots for (A) CD3+CD45+PD-1+ T cells, (B) CD3+CD45+ T cells, (C) CD3+CD4+ or CD3+CD8+ T cells and (D) CD3+CD4+FoxP3+ Tregs. [file 12967_2018_1673_MOESM4_ESM.tif]
